# Impact of the COVID-19 pandemic on mental health and addictions-related visits among citizens of the Métis Nation of Ontario: A repeated cross-sectional study

**DOI:** 10.1371/journal.pmen.0000650

**Published:** 2026-07-14

**Authors:** Noel Tsui, Tera Beaulieu, Abigail J. Simms, Brandon Zagorski, Emily Paterson, Tammy Adams, Sarah A. Edwards, Maria Chiu

**Affiliations:** 1 ICES Central, Toronto, Ontario, Canada; 2 The Métis Nation of Ontario, Ottawa, Ontario, Canada; 3 Weaving Wellness Centre, Toronto, Ontario, Canada; 4 Dalla Lana School of Public Health, University of Toronto, Toronto, Ontario, Canada; PLOS: Public Library of Science, UNITED KINGDOM OF GREAT BRITAIN AND NORTHERN IRELAND

## Abstract

The COVID-19 pandemic led to unprecedented levels of poor mental health, yet Métis-specific experiences remain underreported, despite the Métis being one of the three constitutionally recognized Indigenous Peoples in Canada. This study examined changes in mental health and addictions (MHA)-related outpatient health service utilization among Métis Nation of Ontario (MNO) citizens before and during the COVID-19 pandemic. This was a population-based repeated cross-sectional study. Administrative health data in Ontario, Canada (2017–2022) was linked with the MNO Citizenship Registry (2022). Monthly MHA-related outpatient visit rates were compared between pre-COVID-19 (March 2017-February 2020) and post-COVID-19 (March 2020-December 2022), and rate ratios comparing observed and expected rates were derived using Poisson generalized estimating equations adjusted for age and sex. Stratifications by age and sex were also examined. Among 28,400 MNO citizens, the monthly MHA-related outpatient visit rate was 47.8 per 1000 population pre-COVID-19. During the pandemic, the observed MHA-related outpatient visit rates were 13% higher than expected (95% CI:1.02-1.26). MHA-related outpatient visits post-COVID were higher in females compared to males and MNO citizens aged 65 and older compared to younger citizens. Female MNO citizens aged 65 years and older had the greatest increases in monthly visit rates for mood and anxiety disorders (aRR = 1.45, 95% CI: 1.27-1.65), substance use disorders (aRR = 3.74, 95% CI: 2.03-4.63), and schizophrenia spectrum and other psychotic disorders (aRR = 3.14, 95% CI: 2.00-4.94). In contrast, male MNO citizens aged 65 years and older had the greatest increase in monthly visit rate for other diagnoses (aRR = 1.87, 95% CI: 1.65-2.12). This underscores the importance of high-quality, contemporary data on the mental health outcomes of Métis people to inform the allocation of scarce mental health resources. The findings emphasize the need for targeted mental health interventions, ongoing monitoring, and culturally tailored support to address the mental health needs of the Métis community.

## Introduction

The COVID-19 pandemic and resulting social and economic disruptions have led to unprecedented levels of mental health challenges, such as feelings of uncertainty, anxiety, depression, fear of contracting COVID-19, and worries about economic wellbeing [1–3]. When the pandemic first began, one in five Canadian adults reported moderate to severe symptoms of depression, anxiety, or post-traumatic stress disorder (PTSD), and this prevalence increased to one in four by early 2021 [4]. Although some evidence suggests that population-level mental health worsened early in the pandemic and improved over time as restrictions eased and pandemic-related stressors declined [5], levels of psychological distress remained elevated compared to pre-pandemic levels, potentially due to ongoing factors such as economic instability, healthcare system strain [6], and differences in individual psychological adjustment [5].

Indigenous Peoples were more likely to report moderate to severe symptoms of depression, anxiety, and PTSD than non-Indigenous People [4]. Statistics Canada also conducted a crowdsourcing data collection initiative and found that 60% of Indigenous participants reported worsening mental health since the onset of physical distancing measures, particularly among Indigenous women [1]. During the pandemic, factors such as limited access to services, disruptions to culturally important gatherings, and barriers related to remote and rural living may have contributed to these patterns. These data should be interpreted within the broader context of longstanding mental health disparities between Indigenous and non-Indigenous populations. This context includes the unique experiences of Indigenous Peoples shaped by the historical and ongoing impacts of colonialism, including residential and day schools, unethical medical practices in racially segregated hospitals targeting Indigenous Peoples, past and present child welfare apprehension where Indigenous children were removed from their homes and placed in the care of non-Indigenous families, and forced relocation from historical and ancestral lands [7]. It is also important to note that the COVID-19 pandemic had an intersectional impact, affecting different genders and ages differently, which can be seen reflected in more severe impacts to mental health for some [1]. To date, all mental health reports have been pan-Indigenous based, grouping First Nations, Inuit, and Métis for reporting. It is important to use a distinction-based approach when collecting and reporting data gathered from Indigenous Peoples. Despite some shared experiences in terms of colonization, Indigenous Peoples have their own unique culture, history, languages, and worldviews, which may require different health promotion and intervention strategies. Pan-Indigenous data can obscure important Nation-specific differences, undermine Indigenous self-determination and Nation-specific priorities and ultimately lead to health promotion strategies and services that are not culturally relevant or effective.

With a unique heritage, culture, language, and way of life, Métis are one of three Indigenous Peoples recognized in Canada [8,9]. Their origin stem from the unions of First Nations women and European men, leading to the ethnogenesis of a distinct people over generations [10]. In Ontario, Canada’s most populous province, the Métis Nation of Ontario (MNO), established in 1993, is the only provincially and federally recognized Métis government representing registered Métis in Ontario [11]. The MNO determines citizenship based on the national definition of Métis, which includes: (1) self-identifies as Métis, (2) is distinct from other Aboriginal peoples, (3) is of Historic Métis Nation ancestry, and (4) is accepted by the Métis Nation [10]. The MNO serves over 28,000 Métis citizens across 30 chartered community councils in Ontario, offering more than 30 programs and services [11]. Across Canada, there are several other federally recognized Métis governments in different provinces including the Manitoba Métis Federation, Métis Nation-Saskatchewan, Otipemisiwak Métis Government (formerly Métis Nation of Alberta), and Métis Nation British Columbia.

Despite the significant mental health disparities Indigenous Peoples face, Métis-specific research and data remain limited in scope [12,13]. To address this gap, the MNO has undertaken a broader mixed methods study to gather information about their citizens’ experiences during the COVID-19 pandemic. This paper specifically reports on the quantitative component of the broader study that examined changes in patterns of mental health and addictions (MHA)-related outpatient health service utilization using linked population-based data on MNO citizens before and during the COVID-19 pandemic. By taking a distinction-based approach, this study contributes to a more nuanced understanding of Métis mental health needs and can inform future programing and resources to improve Métis health.

## Methods

### Ethics statement

This study received ethics approval from the University of Toronto Health Sciences Research Ethics Board (#43299). All procedures performed in studies involving human respondents were in accordance with ethical standards of the University of Toronto Health Sciences Research Ethics Board and with the 1964 Helsinki declaration and its later amendments or comparable ethical standards. When registering with the MNO, MNO citizens provide written informed consent for the general use of their information in research and evaluation for the collective benefit of the Métis community. This consent is provided directly by individuals 18 years and older and by parents or legal guardians for children under 18 years of age. This enables the linkage of the MNO Citizenship Registry to ICES data holdings under a Data Governance and Sharing Agreement between the MNO and ICES. Additional consent was not required for participants included in this study as our project was conducted under section 45 of the Ontario’s Personal Health Information Protection Act. ICES is a prescribed entity under section 45 which authorizes ICES to collect and analyze health care and demographic data, without individual consent, for health system evaluation and improvement. All ICES data holdings are fully anonymized for analytical purposes. In addition to adhering to research ethics procedures, this study also adhered to the ethical principles of Métis research. Research on the mental health and wellness was identified as a priority by the MNO. This study was co-developed with MNO staff members, an MNO Senator, MNO citizens, and MNO citizen researchers. It was also reviewed and approved by the Provisional Council of the MNO. The MNO was the primary applicant and held the research funds for the project. Results were co-interpreted with MNO staff members and citizens and shared back with MNO elected officials and citizens for iterative feedback and refinement before publication.

### Study design

A population-based, repeated cross-sectional study of all MNO citizens living in Ontario, Canada was conducted between January 1, 2017, to December 31, 2022. This period allowed for multiple pre-pandemic years (2017–2019) to account for potential year-to-year variability and mirrored the available pandemic period (2020–2022) at the time of analyses. Additionally, the pandemic period analysed included both the acute and longer-term mental health impacts of COVID-19. Since 2009, the MNO and ICES (formerly the Institute for Clinical Evaluative Sciences) have collaborated to utilize the administrative health data available to ICES by linking it with the MNO Citizenship Registry through a data governance and sharing agreement [14]. ICES is an independent, non-profit research institute that is legally permitted under Ontario’s health information privacy law to collect and analyze health care and demographic data, without consent, for the purpose of evaluating and improving the health system. All projects intending to use the MNO Citizenship Registry or explore Métis health at ICES must be reviewed and approved by the MNO before advancing through ICES’s approval procedures. The MNO Citizenship Registry was linked to administrative health data through an ICES Key Number (IKN). Each person accessing healthcare in Ontario has been assigned a unique IKN by ICES based on their direct personal identifiers; these identifies are removed from the data after IKN has been assigned to ensure individual-level privacy and confidentiality while retaining the ability to deterministically link across datasets held in ICES [15].

All MNO citizens registered as of November 2022 were linked using the IKN to the Registered Persons Database (RPDB); a database of Ontario’s health insurance registrants living in Ontario, Canada available at ICES. MNO citizens older than 105, and/or with missing age, sex, or postal code were excluded. Socio-demographic variables including age, sex (female, male), income quintiles, and rurality (rural, urban) were extracted from RPDB. Age and sex were controlled in the analysis and income quintile and rurality were only described due to low sample sizes.

Using IKN, we also linked MNO citizens to their Ontario Health Insurance Plan (OHIP), a physician billings database, to identify MHA-related visits to family physicians, pediatricians, psychiatrists, and nurse practitioners. Physicians’ services reimbursed by the Ministry of Health, either by a fee for service claim or shadow billed from those in a capitation practice, will be in the OHIP dataset. Data were accessed for research purposes between 08/02/2023 and 28/06/2023.

### Outcome measures

Our main outcome was the number of MHA-related outpatient visits to a pediatrician, family physician, psychiatrist, or nurse practitioners. These included both in-person or virtual outpatient visits. Visits were classified into broad diagnostic groupings based on the diagnostic code on the OHIP record including mood and anxiety disorders (OHIP diagnostic codes: 296, 300, 311), substance use disorders (OHIP diagnostic codes: 291, 292, 303, 304), schizophrenia spectrum and other psychotic disorders (OHIP diagnostic codes: 295, 297, 298), and other diagnoses (Behavioural and neuro-development disorders – OHIP diagnostic codes: 299, 313, 314, 315; Non-categorized mental health disorders – OHIP diagnostic codes: 301, 302, 306, 307, 309; Social problems – OHIP diagnostic codes: 899, 902, 904, 909; Other social and family problems – OHIP diagnostic codes: 897, 898, 900, 901, 905, 906). A visit (i.e., physician encounter) was restricted to one per patient per physician per day. Repeated cross-sectional analyses of aggregate MHA-related outpatient visit rates were reported per 1000 MNO eligible citizens, overall and stratified by age group (<29, 30–64, 65+), sex (female, male), and diagnostic group (mood and anxiety disorders, substance use disorders, schizophrenia spectrum and other psychotic disorders, and other diagnoses) for each month from January 1, 2017, to December 31, 2022.

### Statistical analyses

To measure changes in outpatient mental health care use, we used Poisson Generalized Estimating Equations (GEE) models for clustered count data as previously used in a similar study [16]. This approach accounts for correlation in repeated responses over time within the same region and allows for time-dependent response rates. Unlike time series models, GEE models produce consistent estimates of regression coefficients and standard errors, even when the correlation structure is mis-specified. Our Poison GEE model also provided the desired population-average estimates, so the pre/post-COVID effect can be estimated at the age group and sex cluster level. Pre-existing MHA-related outpatient visit utilization variation was estimated using a full 36 months prior to the start of the pandemic and our plots suggest that the model sufficiently predicted the observed utilization. Standard residual diagnostics are not applicable to Poisson GEE models. There were no missing data in the dataset used for the GEE model.

Briefly, data were aggregated into age, sex, and monthly rates and 3-year pre–COVID-19 trends were used to forecast expected post–COVID-19 trends in the absence of the pandemic. The pre–COVID-19 model included age- and sex-specific indicators, a continuous weekly time variable starting January 1, 2017, to capture trends in visit rates through February 29, 2020, and monthly indicators (with April as the reference month) to account for seasonal variation. We estimated the expected post–COVID-19 visit rates and 95% Confidence Intervals (CIs) by applying the pre–COVID-19 regression coefficients to the post–COVID-19 age-sex-month strata and exponentiating. The relative change in post–COVID-19 visit rates was calculated as the ratio of observed to expected rates, adjusted for age and sex; these were done by calculating the difference between observed and expected log rates, estimating standard errors and 95% CIs for this difference as it was a linear combination of regression coefficients, and then exponentiating to present rates and 95% CIs on the original scale. Sex and age group stratified rates and rate ratios in the post-COVID-19 period were calculated using the weighted mean of the monthly predicted estimates.

## Results

### Sample

The MNO Citizenship Registry included 29,319 citizens in November 2022. There was a total of 28,400 MNO citizens who met the criteria for inclusion in our study cohort (see S1 Table); The mean age of our cohort was 43.5 years, 50.3% were male, 28.3% lived in rural areas, and 17.3% and 20.8% lived in the lowest and highest area-level income quintiles, respectively (Table 1).

**Table 1:** Descriptive characteristics and pre-COVID mental health visits for 28,400 citizens of the Métis Nation of Ontario included in the analyses.

Descriptive characteristics		n (%)
Age	Mean (SD)	43.5 (21.1)
	Median (Q1-Q3)	44 (27-60)
	Min – Max	0 – 101
	Missing (%)	0 (0.0%)
		
Age Group (years)	≤29	7,971 (28.1%)
	30-64	15,408 (54.3%)
	65-105	5,021 (17.7%)
	Missing	0 (0.0%)
		
Sex	Females	14,112 (49.7%)
	Males	14,288 (50.3%)
	Missing	0 (0.0%)
		
Rurality	Rural	8,041 (28.3%)
	Urban	19,505 (68.7%)
	Missing	854 (3.0%)
		
Neighbourhood Income Quintile	1-Lowest	4,919 (17.3%)
	2	5,765 (20.3%)
	3	5,348 (18.8%)
	4	5,603 (19.7%)
	5-Highest	5,907 (20.8%)
	Missing	858 (3.0%)
		
At least one mental health visits (pre-COVID-19)		
	Type of mental health visit (pre-COVID-19)	8,833 (31.1%)
	Mood and anxiety disorders	7,022 (24.7%)
	Schizophrenia spectrum and other psychotic disorders	171 (0.6%)
	Substance use disorders	605 (2.1%)
	Other diagnosis*	3,144 (11.1%)
	Behavioural and neuro-developmental disorders	830 (3.0%)
	Non-categorized mental health disorders (personality disorders, sexual deviations, psychosomatic disturbances, habit spasms/tics/stuttering/tension headaches/anorexia nervosa/sleep disorders/enuresis, adjustment reaction)	2042 (7.2%)
	Social problems	302 (1.1%)
	Other social and family problems	298 (1.0%)

*Categories under ‘Other diagnosis’ are not mutually exclusive

### Changes in MHA-related outpatient health service utilization

**Fig 1:**
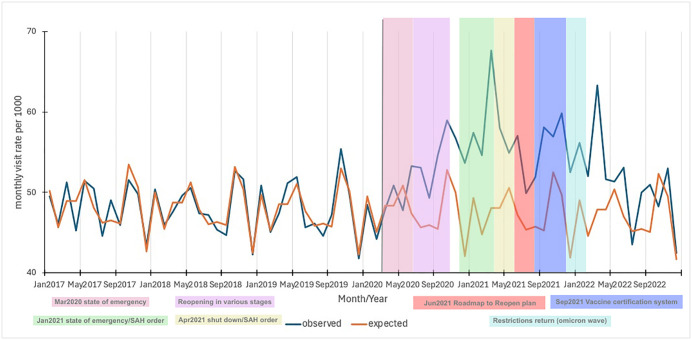
Observed and expected mental health and addictions-related outpatient monthly visit rates among citizens of the MNO (N = 28,400) from January 2017 to December 2022.

In the pre-COVID-19 period (January 1, 2017 – February 29, 2020), the monthly visit rates for all MHA-related outpatient visits were 47.4 per 1000 population and rose to 52.8 per 1000 population during the COVID-19 pandemic period. Fig 1 shows the observed and expected monthly outpatient visit rates before and during the COVID-19 pandemic. For overall MHA-related outpatient visits, the monthly visit rate was 13% higher than expected (53.5 vs 47.4/1000 population, adjusted relative rate [aRR]= 1.13; 95% CI: 1.03-1.25).

### Stratified analyses by age, sex, and diagnostic type

Female MNO citizens and citizens 65 years and older had overall MHA-related monthly visit rates that were 18% and 47% higher than expected. Among male MNO citizens and certain age groups (≤29 and 30–64 years), the overall MHA-related monthly visit rates ranged between 7% to 11% higher than expected. The increase in overall MHA-related monthly visit rates was highest among female MNO citizens who were 65 years and older with rates 54% higher than expected (95% CI: 1.39-1.69). Overall the monthly visit rate for mood and anxiety disorders was 12% higher (32.0 vs 28.5/1000 population, RR = 1.12; 95% CI: 0.98-1.28), schizophrenia spectrum and other psychotic disorders was 9% higher (1.0 vs 0.9/1000 population, aRR = 1.09; 95% CI: 0.70-1.72), substance use disorders was 2% higher (10.9 vs 10.7/1000 population, aRR = 1.02; 95% CI: 0.82-1.26), and other diagnoses was 35% higher than expected (10.2 vs 7.5/1000 population, aRR = 1.35; 95% CI: 1.19-1.53), although these did not reach statistical significance.

**Fig 2:**
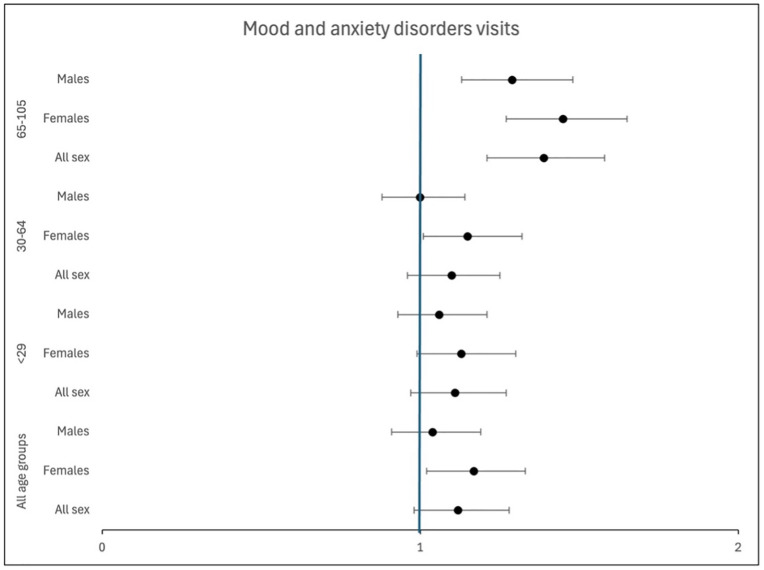
Age and sex stratified rates of outpatient mental health visits for mood and anxiety disorders in citizens of the Métis Nation of Ontario following the onset of the COVID-19 pandemic in Ontario, Canada.

For mood and anxiety disorder visits (Fig 2), the monthly visit rates among female MNO citizens and MNO citizens 65 years and older were 17% and 39% higher than expected. The rates among male MNO citizens and the other age groups (≤29 and 30–64) ranged between 4% to 11% higher than expected. The increase in monthly visit rate for mood and anxiety disorders was highest for female MNO citizens who were 65 years and older, where it was 45% higher than expected.

**Fig 3:**
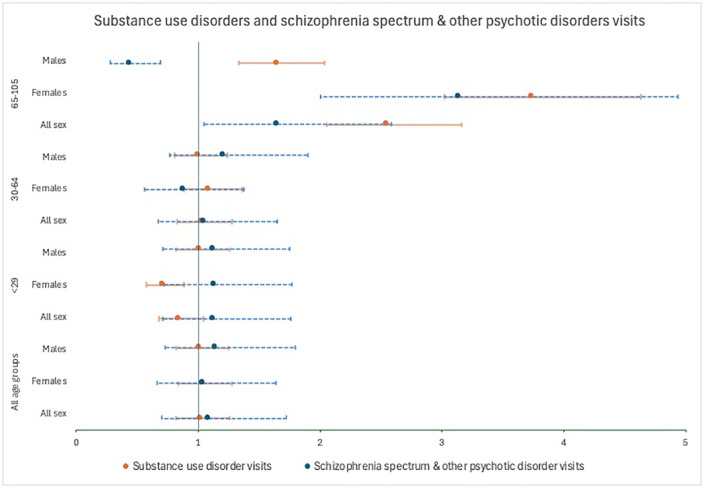
Age and sex stratified rates of outpatient mental health visits for substance use disorders and schizophrenia spectrum and other psychotic disorders in citizens of the Métis Nation of Ontario following the onset of the COVID-19 pandemic in Ontario, Canada.

For substance use disorder visits (Fig 3), a significant increase in the monthly visit rate was found among female MNO citizens 65 years or older. The monthly visit rate for substance use disorders among MNO citizens aged 29 or younger was lower than expected. Meanwhile, the monthly visit rate for substance use disorders among male MNO citizens and other age groups (30–64 and 65+) were on par with the expected rates.

For schizophrenia spectrum and other psychotic disorder visits (Fig 3), a significant increase in the monthly visit rate was found among female MNO citizens 65 years and older. Considering all age groups, the monthly visit rate for schizophrenia spectrum and other psychotic disorders among male MNO citizens was 14% higher than expected, with those aged 65 years or older showing the highest increase at 65% higher than expected.

**Fig 4:**
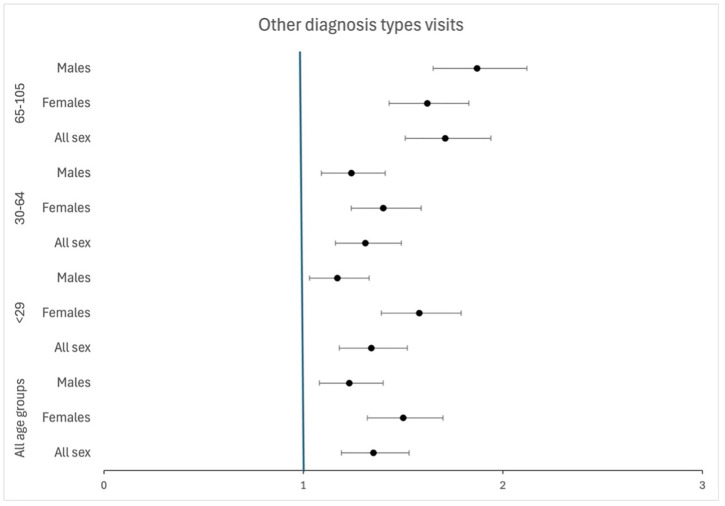
Age and sex stratified rates of outpatient mental health visits for other diagnosis types (Behavioural and neuro-developmental disorders, non-categorized mental health disorders, social problems, other social and family problems) in citizens of the Métis Nation of Ontario following the onset of the COVID-19 pandemic in Ontario, Canada.

For monthly visit rates for all the other diagnostic groups (Fig 4), the largest increase was among male MNO citizens aged 65 years and older. Female MNO citizens had 50% higher than expected visit rates for other diagnostic groupings. MNO citizens aged 65 years and older had 71% higher than expected visits rates.

## Discussion

To our knowledge, this is the first population-based linkage study to explore the impact of the COVID-19 pandemic on Métis people’s MHA-related outpatient visits. The results, limited to Ontario, showed that MNO citizens experienced a significant increase in the rate of MHA-related outpatient visits, with rates 13% above the expected levels for the 33 months following the onset of the COVID-19 pandemic. This result is consistent with other studies examining the MHA-related service utilization after the onset of the COVID-19 pandemic [16,17]. For example, increases of 10–15% have been reported among the Ontario youth population [16]. In contrast, a more modest increase of approximately 3% has been observed in the general Canadian population, while trends across other high-income countries have varied [17].

We also observed sex- and age-based differences in MHA-related visits following the pandemic onset compared to the expected levels. Female MNO citizens and MNO citizens 65 years and older had the highest rates of outpatient visits. This finding aligns with Arriagada et al. (2020), where a higher percentage of Indigenous women reported symptoms consistent with moderate or severe generalized anxiety and reported their mental health had worsened since the start of the pandemic compared to Indigenous men. This finding may be explained by the increased caregiving and household responsibilities placed on women during the pandemic along with increased pandemic-related social isolation which would have impacted Métis kin and community connections [18]. Even before the pandemic, Métis women noted they did most of the unpaid work in both the home and community [19]. Qualitative findings from the other component of the mixed methods study (unpublished) conducted by the MNO exploring mental wellness among MNO citizens during the COVID-19 pandemic, suggested that Métis mothers reported that their children’s mental health struggles affected their own mental health, particularly due to isolation during stay-at-home orders. This is consistent with what has been documented historically about the importance of the roles women have played in Métis communities [20]. Similar trends were observed in the Canadian population, where women were less likely to report better mental health since the onset of the pandemic [3,21] and were more likely to report moderate to severe generalized anxiety disorder than men [22]. Furthermore, Indigenous Peoples and older adults are considered a high-risk group for experiencing social isolation during the pandemic [23]. This may help explain the higher rates of MHA-related visits observed in our study, as older MNO citizens may have experienced disruptions to community support and health services, reduced participation in social gatherings or employment due to fear of contracting COVID-19 or public health measures, and challenges accessing internet or using online tools to stay connected with loved ones [23]. However, this finding contrasts with Nwachukwu et al. (2020) [24], which found that younger participants in Alberta (aged 25 or younger) had the highest prevalence in high/moderate stress, anxiety, and depression during the pandemic. These discrepancies highlight the complexity and variability of the impact of the COVID-19 pandemic on mental health and wellness within different populations and provinces. Furthermore, it emphasizes the need for local data and tailored health promotion and intervention strategies.

For mood and anxiety disorder visits, the overall monthly visit rate was higher than expected. This finding aligns with Burnett et al. (2022) [25], where an online survey distributed to Indigenous communities in Canada in 2020 found that 40% of respondents reported symptoms of depression and 45% reported symptoms of anxiety following the onset of the pandemic. Additionally, a national survey conducted in April 2020 revealed that the proportion of Canadians reporting high to extremely high anxiety quadrupled, and those reporting high depression doubled since the onset of the COVID-19 pandemic [26].

For substance use disorder visits, most monthly visit rates were on par with the expected rates, except for a significant increase in female MNO citizens 65 years or older and a decrease in MNO citizens aged 29 or younger. Although these findings contrast with some studies that found Indigenous youth [27] and Canadian youth [21] used more substances during the height of the pandemic compared to before, they were in line with other studies that suggested lower substance use in adolescents and young adults as these behaviours are influenced by peer interaction, social context, and access for younger age groups [28,29]. This contrasts with older age adults where stress-related and solitary substance use may be more common. In general, older populations had lower odds of increased alcohol and cannabis use [21]. However, heightened alcohol and cannabis use were observed among Canadians [22,26], with women reporting a greater increase in cannabis use than men [21]. Increased substance use may be one reason for the increase in MHA-related visits for Métis women over 65 years; however, this may also be because women are more likely to have frequent contact with and higher attachment to primary care providers [30,31]. Furthermore, for Métis women in particular, they may be more likely to have frequent outpatient visits for chronic disease management and medication monitoring as chronic diseases are more prevalent in Métis [32,33], which would increase opportunities for the identification and management of substance-related concerns. Although Métis men also have increased prevalence of chronic conditions, they may be less likely to engage in outpatient visits [34].

For schizophrenia spectrum and other psychotic disorder visits, MNO citizens aged 65 and older had the most significant increase in monthly visit rate. Currently, no studies report on psychotic disorder visit rates among Indigenous populations in Canada. However, a 9% increase in adult hospitalizations for psychotic disorders [35] and a 66% increase in adolescent inpatient psychosis admissions [36] were observed in Ontario, Canada during the pandemic. The slightly greater increase in visit rates among male citizens, in contrast to other disorders where increases are mainly seen in female citizens, may be explained by the higher incidence of schizophrenia in men [37]. This effect may have been further amplified by pandemic-related loneliness, stressful life events (e.g., job loss or loss of loved ones), and reduced family or social support, which are known psychosocial factors that contribute to the development of psychosis [37].

For all other diagnoses (behavioural and neuro-developmental disorders, non-categorized mental health disorders (e.g., personality disorders, sexual deviations, psychosomatic disturbances, habit spasms/tics/stuttering/tension headaches/anorexia nervosa/sleep disorders/enuresis, and adjustment reactions), social problems, and other social and family problems), all monthly visit rates were higher than expected, with MNO citizens aged 65 years and older experiencing the most significant increase. No specific data on visit rates among Indigenous populations were found. However, increases in the rates of Canadians self-reporting or meeting the criteria for obsessive compulsive disorders [38] and sleeping disorders [39] were observed during the pandemic. Furthermore, hospital admissions for eating disorders and personality disorders increased substantially during the pandemic [40]. Post COVID-19 conditions (long-COVID) also commonly involve neuropsychiatric symptoms, such as difficulty thinking and solving problems (brain fog), stress or anxiety, headache, and sleep disturbances, with older adults reporting the highest rate of these symptoms following COVID-19 infection [41,42]. This may have contributed to the significant increase in visit rates among MNO citizens aged 65 years and older for this diagnostic group. However, we did not examine whether citizens had previously contracted COVID-19 before or experienced long-COVID symptoms, and this should be explored in future research.

### Strengths and limitations

A major strength of this study is that it is the first to use a distinctions-based approach to examine the mental health impact of the COVID-19 pandemic among a Métis population. These results can inform MNO leadership and policymakers on mental health resource allocation and promotion strategies specifically targeting women and senior citizens to improve mental health outcomes and identify the need to advocate for increased mental health resources for MNO citizens during public health emergencies. This data also helps fill the research gap on the impact of the COVID-19 pandemic on mental health among Indigenous populations. In addition, as an MNO-led project, it supports and upholds Indigenous data sovereignty by ensuring that the MNO has control and decision-making power over how data about them is collected, stored, accessed, and used [43].

Our study used a population-based approach linking administrative health data, which has numerous advantages including population coverage, ability to link with other data sources, and limited selection bias [44,45]. Administrative health data sources also have the limitation that variables included are not collected for research purposes, which results in missing information [45]. For our study, we did not have access to individual level socioeconomic status variables, like income, education, and employment or more wholistic health variables included in the Métis definition of health, such as connections to kin, community, lands, and waters [46].We were also limited to biological sex and did not have gender available to analyze.

This study also has limitations. First, in terms of our study population, the MNO Citizenship Registry only includes Métis people who meet the eligibility criteria to be a Métis citizen [10,47] and who have chosen to register with the MNO. We were unable to include Métis registered with other Métis governments who are currently living in Ontario, MNO citizens who may be accessing healthcare services federally as members of the Canadian Armed Forces, or Métis people who would meet eligibility criteria to become an MNO citizen but have not chosen to register. Therefore, the study results only represent MNO citizens accessing Ontario healthcare rather than all Métis living in Ontario. Second, in terms of capturing the full impact of the pandemic on MNO citizens mental health and addictions-related outpatient visits, we were limited to accessing publicly funded care and were not able to report either unmet needs for MNO citizens or MNO citizens who may have accessed non-physician-based MHA-related care privately. Third, our diagnostic groupings represent broad categories based on physician billing codes and health service use, rather than clinical diagnostic criteria, which may obscure trends in specific conditions. Furthermore, we were unable to disaggregate the individual diagnoses within the “other diagnoses” category or report by area-level income quintile and rurality due to small cell counts and the risk of re-identification. The qualitative component of the mixed-method study that the MNO conducted (not reported in this paper), which included sharing circles and 1–1 interviews with MNO citizens, will address some of these limitations by providing more in-depth insights into mental health challenges and coping strategies MNO citizens experienced during the COVID-19 pandemic.

## Conclusion

This is the first Métis-specific study to our knowledge to explore the impact of the COVID-19 pandemic on MHA-related outpatient visits. Our findings indicate that the MNO citizens experienced an increase in MHA-related outpatient visits during the COVID-19 pandemic, with the highest increase seen in women and citizens 65 years and older. These results offer high-quality, contemporary information on the mental health impacts from the COVID-19 pandemic which is crucial to inform the allocation of scarce mental health resources now and in future public health emergencies. In particular, these findings reinforce the need for distinctions‑based Indigenous health policies that account not only for differences between Indigenous Peoples, but also for age‑ and gender‑specific patterns of healthcare use within Nations. Strengthening Métis‑specific data systems and integration of Métis-led research into provincial health planning may support more responsive and effective service delivery. Continued monitoring of MHA-related outpatient visits in MNO citizens is warranted and ongoing research will report on the stories and experiences of MNO citizens using qualitative data. Future research will examine additional self-reported measures of mental health by MNO citizens during the COVID-19 pandemic like loneliness and explore the feasibility of reporting specific mental health conditions beyond broad diagnostic groupings.

## Supporting information

S1 Table Cohort exclusion criteria for citizens of the Métis Nation of Ontario (MNO). (DOCX)

## References

[1] Arriagada P, Hahmann T, O’donnell V. Indigenous people and mental health during the COVID-19 pandemic. 2020.

[2] Galea S, Merchant RM, Lurie N. The mental health consequences of COVID-19 and physical distancing: The need for prevention and early intervention. JAMA Internal Medicine. 2020;:817–8. doi: 10.1001/jamainternmed.2020.1562 32275292

[3] Findlay LC, Arim R, Kohen D. Understanding the Perceived Mental Health of Canadians During the COVID-19 Pandemic. Health Rep. 2020;31(4):22–7. doi: 10.25318/82-003-x202000400003-eng 32644764

[4] Statistics Canada. Survey on COVID-19 and Mental Health, February to May 2023. 2023. https://www150.statcan.gc.ca/n1/daily-quotidien/231212/dq231212c-eng.htm

[5] Zrnić Novaković I, Ajduković D, Ajduković M, Kenntemich L, Lotzin A, Schäfer I, et al. Mental health during and after the COVID-19 pandemic - a longitudinal study over 42 months in five European countries. Eur J Psychotraumatol. 2025;16(1):2488700. doi: 10.1080/20008066.2025.2488700 40260985 PMC12016253

[6] Ding W, Zhang Y, Wang M-Z, Wang S. Post-pandemic mental health: Understanding the global psychological burden and charting future research priorities. World J Psychiatry. 2025;15(10):109502. doi: 10.5498/wjp.v15.i10.109502 41112617 PMC12531953

[7] Matheson K, Seymour A, Landry J, Ventura K, Arsenault E, Anisman H. Canada’s Colonial Genocide of Indigenous Peoples: A Review of the Psychosocial and Neurobiological Processes Linking Trauma and Intergenerational Outcomes. International Journal of Environmental Research and Public Health. 2022. doi: 10.3390/ijerph19116455 PMC9179992 35682038

[8] Métis Nation of O. Who are the Métis?. https://www.metisnation.org/culture-heritage/who-are-the-metis/?doing_wp_cron=1715787696.4315259456634521484375

[9] Government of Canada. INAN - Section 35 of the Constitution Act 1982 - Background. 2021. Accessed 2024 July 30. https://www.canada.ca/en/immigration-refugees-citizenship/corporate/transparency/committees/inan-jan-28-2021/inan-section-35-consitution-act-1982-background-jan-28-2021.html

[10] Métis Nation of Ontario. Citizenship. Accessed 2025 April 1. https://www.metisnation.org/registry/citizenship/

[11] Métis Nation of Ontario. About the MNO. Accessed 2024 May 14. https://www.metisnation.org/about-the-mno/

[12] Kumar MB, Wesche S, McGuire C. Trends in Métis-related health research (1980-2009): identification of research gaps. Can J Public Health. 2012;103(1):23–8. doi: 10.1007/BF03404064 22338324 PMC6974209

[13] Macdougall B. Land, family and identity: contextualizing Metis health and well-being. Prince George: National Collaborating Centre for Aboriginal Health; 2017.

[14] Pyper E, Henry D, Yates EA, Mecredy G, Ratnasingham S, Slegers B, et al. Walking the Path Together: Indigenous Health Data at ICES. Healthcare Quarterly. 2018;20:6–9. doi: 10.12927/hcq.2018.25431 29595420

[15] ICES. Working with ICES Data.

[16] Saunders NR, Kurdyak P, Stukel TA, Strauss R, Fu L, Guan J, et al. Utilization of Physician-Based Mental Health Care Services Among Children and Adolescents Before and During the COVID-19 Pandemic in Ontario, Canada. JAMA Pediatr. 2022;176(4):e216298. doi: 10.1001/jamapediatrics.2021.6298 35129604 PMC8822447

[17] Silva-Valencia J, Lapadula C, Westfall JM, Gaona G, de Lusignan S, Sarkadi Kristiansson R. Effect of the COVID-19 pandemic on mental health visits in primary care: an interrupted time series analysis from nine INTRePID countries. 2024. 10.1016/j.eclinm.2024.102533 PMC10940140 38495523

[18] Flores J, Emory K, Santos X, Mashford-Pringle A, Barahona-Lopez K, Bozinovic K. I think the mental part is the biggest factor: An exploratory qualitative study of COVID-19 and its negative effects on Indigenous women in Toronto, Canada. Frontiers in Sociology. 2022;7. doi: 10.3389/fsoc.2022.790397 PMC9108416 35586263

[19] Les Femmes Michif Otipemisiwak. Building a Métis Women’s Blueprint. Les Femmes Michif Otipemisiwak; 2023.

[20] Women of the Métis Nation. Traditional Knowledge Policy Paper.

[21] Brotto LA, Chankasingh K, Baaske A, Albert A, Booth A, Kaida A, et al. The influence of sex, gender, age, and ethnicity on psychosocial factors and substance use throughout phases of the COVID-19 pandemic. PLoS One. 2021;16(11):e0259676. doi: 10.1371/journal.pone.0259676 34807908 PMC8608308

[22] Lin SL. Generalized anxiety disorder during COVID-19 in Canada: Gender-specific association of COVID-19 misinformation exposure, precarious employment, and health behavior change. J Affect Disord. 2022;302:280–92. doi: 10.1016/j.jad.2022.01.100 35093413 PMC8799934

[23] Wister A, Kadowaki L. Social isolation among older adults during the pandemic. Employment and Social Development Canada. 2021.

[24] Nwachukwu I, Nkire N, Shalaby R, Hrabok M, Vuong W, Gusnowski A, et al. COVID-19 pandemic: age-related differences in measures of stress, anxiety and depression in Canada. Int J Environ Res Public Health. 2020;17(17):6366. doi: 10.3390/ijerph17176366 32882922 PMC7503671

[25] Burnett C, Purkey E, Davison CM, Watson A, Kehoe J, Traviss S. Spirituality, community belonging, and mental health outcomes of indigenous peoples during the COVID-19 pandemic. Int J Environ Res Public Health. 2022;19. doi: 10.3390/ijerph19042472 PMC8872600 35206662

[26] Dozois DJA. Anxiety and depression in Canada during the COVID-19 pandemic: a national survey. Canadian Psychology. 2021;62:136–42. doi: 10.1037/cap0000251

[27] Mollons MO, Penner KE, Elsom AL, Cameron EE, Hunter S, Woods L, et al. COVID-19 and indigenous youth wellbeing: A review. Curr Opin Psychol. 2023;53:101659. doi: 10.1016/j.copsyc.2023.101659 37597427

[28] Romano I, Patte KA, de Groh M, Jiang Y, Leatherdale ST. Perceptions of and adherence to early COVID-19-related restrictions and associations with substance use among youth in Canada. Health Promot Chronic Dis Prev Can. 2022;42(11–12):479–89. doi: 10.24095/hpcdp.42.11/12.03 36165768 PMC9903852

[29] Miech RA. The intensity of adolescent substance use before and after the COVID-19 pandemic. Am J Prev Med. 2026;70:108166. doi: 10.1016/j.amepre.2025.108166 41135919 PMC12616519

[30] Government of Canada. Health of Canadians: Access to health care. 2025. https://www150.statcan.gc.ca/n1/pub/82-570-x/2024001/section4-eng.htm

[31] Bayoumi I, Glazier RH, Jaakkimainen L, Premji K, Kiran T, Frymire E, et al. Trends in attachment to a primary care provider in Ontario, 2008-2018: an interrupted time-series analysis. CMAJ Open. 2023;11(5):E809–19. doi: 10.9778/cmajo.20220167 37669813 PMC10482493

[32] Métis Nation of Ontario. Chronic disease and risk factors in the Métis population of Ontario. Métis Nation of Ontario. 2015. https://www.cancercare.on.ca

[33] Martens P, Bartlett JG, Burland EMJ, Prior HJ, Burchill CA, Huq S. Profile of Metis health status and healthcare utilization in Manitoba: a population-based study. Manitoba Centre for Health Policy in collaboration with the Manitoba Metis Federation; 2012.

[34] Thompson AE, Anisimowicz Y, Miedema B, Hogg W, Wodchis WP, Aubrey-Bassler K. The influence of gender and other patient characteristics on health care-seeking behaviour: a QUALICOPC study. BMC Fam Pract. 2016;17:38. doi: 10.1186/s12875-016-0440-0 27036116 PMC4815064

[35] Tanner B, Kurdyak P, de Oliveira C. Adult Psychiatric Hospitalizations in Ontario, Canada Before and During the COVID-19 Pandemic. Can J Psychiatry. 2023;68(12):925–32. doi: 10.1177/07067437231167386 37006178 PMC10657583

[36] Deren B, Matheson K, Cloutier P. Rate of adolescent inpatient admission for psychosis during the COVID-19 pandemic: a retrospective chart review. Early Interv Psychiatry. 2023;17(1):115–7. doi: 10.1111/eip.13316 35689347 PMC9349685

[37] Li X, Zhou W, Yi Z. A glimpse of gender differences in schizophrenia. Gen Psychiatr. 2022;35(4):e100823. doi: 10.1136/gpsych-2022-100823 36118418 PMC9438004

[38] Abba-Aji A, Li D, Hrabok M, Shalaby R, Gusnowski A, Vuong W, et al. COVID-19 Pandemic and Mental Health: Prevalence and Correlates of New-Onset Obsessive-Compulsive Symptoms in a Canadian Province. Int J Environ Res Public Health. 2020;17(19):6986. doi: 10.3390/ijerph17196986 32987764 PMC7579625

[39] Shillington KJ, Vanderloo LM, Burke SM, Ng V, Tucker P, Irwin JD. Not so sweet dreams: adults’ quantity, quality, and disruptions of sleep during the initial stages of the COVID-19 pandemic. Sleep Med. 2022;91:189–95. doi: 10.1016/j.sleep.2021.02.028 33685852 PMC9017869

[40] Patten SB, Dimitropoulos G, Williams JVA, Rao S, Fahim M, Sharifi V. Hospital admissions for personality disorders increased during the COVID-19 pandemic. Can J Psychiatry. 2023;68:470. doi: 10.1177/07067437231155999 36786026 PMC9931879

[41] 41.Government of Canada. Post COVID-19 condition (long COVID): Symptoms and treatment. 2026. Accessed 2026 May 12.

[42] Government of Canada. Long-term neuropsychiatric symptoms of COVID-19 among adults. 2024. https://health-infobase.canada.ca/datalab/post-covid-condition-neuropsychiatric-symptoms.html

[43] Kukutai T, Taylor J. Data sovereignty for indigenous peoples: current practice and future needs. Indigenous Data Sovereignty. ANU Press. 2016. doi: 10.22459/caepr38.11.2016.01

[44] Virnig BA, McBean M. Administrative data for public health surveillance and planning. Annu Rev Public Health. 2001;22:213–30. doi: 10.1146/annurev.publhealth.22.1.213 11274519

[45] Thygesen LC, Ersbøll AK. When the entire population is the sample: strengths and limitations in register-based epidemiology. Eur J Epidemiol. 2014;29(8):551–8. doi: 10.1007/s10654-013-9873-0 24407880

[46] Métis Nation Council. Métis nation: a vision for health. 2023. https://www.metisnation.ca/uploads/documents/3-1)Me%CC%81tis%20Vision%20for%20Health-July%2012%20update.pdf

[47] Métis Nation of Ontario. Métis Registry & Citizenship. https://www.metisnation.org/registry/

