# Supplementary material for: Impact of the COVID-19 pandemic on mental health and addictions-related visits among citizens of the Métis Nation of Ontario: A repeated cross-sectional study
Source: PLOS Ment Health. 2026 Jul 14;3(7):e0000650. doi: 10.1371/journal.pmen.0000650 (PMC13367706; doi:10.1371/journal.pmen.0000650)
Supplement: S1 Table — (DOCX) [file pmen.0000650.s001.docx]

Supplementary

S1 Table. Cohort exclusion criteria for citizens of the Métis Nation of Ontario (MNO).

| **Exclusion** | **N (%)** |
| --- | --- |
| MNO registrants with a valid Ontario health card number | N=29,319 |
| Died < Jan 1, 2017 | 312 (1.1%) |
| Age >105 years | 18 (0.1%) |
| Non-Ontario postal code | 570 (1.9%) |
| Missing age and/or sex | 22 (0.1%) |
| Any of the above exclusions | 919 (3.1%) |
| Study cohort: MNO registrants who met the inclusion criteria | 28,400 (96.9%) |
