# Supplementary material for: Impact of the COVID-19 pandemic on mental health and addictions-related visits among citizens of the Métis Nation of Ontario: A repeated cross-sectional study
Source: PLOS Ment Health. 2026 Jul 14;3(7):e0000650. doi: 10.1371/journal.pmen.0000650 (PMC13367706; doi:10.1371/journal.pmen.0000650)
Supplement: Attachment — Submitted filename: Response table 20260526.docx [file pmen.0000650.s003.docx]

| Editor’s comments | Responses |
| --- | --- |
| 1. We noticed you have some minor occurrence of overlapping text with the following previous publication(s), which needs to be addressed:  - DOI 10.3389/978-2-8325-3976-7 - doi:10.1001/jamapediatrics.2021.6298  In your revision ensure you cite all your sources (including your own works), and quote or rephrase any duplicated text outside the methods section. Further consideration is dependent on these concerns being addressed. | Thank you for noting this. We carefully reviewed our manuscript for potential text overlap with the following publications.  For doi:10.1001/jamapediatrics.2021.6298, we identified overlapping phrasing in the methods section and have revised the text throughout the manuscript to ensure appropriate paraphrasing and proper citation where relevant.  For DOI 10.3389/978-2-8325-3976-7, we were unable to identify any overlapping text with our manuscript. We would appreciate any additional guidance if specific sections of concern can be identified. |
| 2. Please amend your detailed Financial Disclosure statement. This is published with the article. It must therefore be completed in full sentences and contain the exact wording you wish to be published.  i. State the initials, alongside each funding source, of each author to receive each grant. For example: "This work was supported by the National Institutes of Health (####### to AM; ###### to CJ) and the National Science Foundation (###### to AM)." ii. State what role the funders took in the study. If the funders had no role in your study, please state: “The funders had no role in study design, data collection and analysis, decision to publish, or preparation of the manuscript.” | The financial disclosure statement has been revised accordingly. |
| 3. Please ensure that your Ethics Statement is available in its entirety at the beginning of your Methods section, under a subheading 'Ethics Statement'. | Ethics Statement has been moved to the beginning of the Methods section. |
| 4. Please provide separate figure files in .tif or .eps format. | All figure files have been converted to .tif format |
| 5. We have noticed that you have uploaded Supporting Information files, but you have not included a list of legends. Please add a full list of legends for your Supporting Information files after the references list. | Thank you for the comment. We have added a ‘Supporting Information Legends’ section after the references list. |
| 6. For studies involving third-party data, we encourage authors to share any data specific to their analyses that they can legally distribute. PLOS recognizes, however, that authors may be using third-party data they do not have the rights to share. When third-party data cannot be publicly shared, authors must provide all information necessary for interested researchers to apply to gain access to the data. (https://journals.plos.org/plosone/s/data-availability#loc-acceptable-data-access-restrictions)    For any third-party data that the authors cannot legally distribute, they should include the following information in their Data Availability Statement upon submission:   1) A description of the data set and the third-party source  2) If applicable, verification of permission to use the data set  3) Confirmation of whether the authors received any special privileges in accessing the data that other researchers would not have  4) All necessary contact information others would need to apply to gain access to the data | This study did not use third-party data. |

| Reviewer #1’s comments | Responses |
| --- | --- |
| 1. Abstract: lines 33-35 and 35-36 repeat the same findings of sex and age disparities in MHA visits. | Thank you for catching this, the abstract has been revised. |
| 2. How is this study being conducted in relation to MNO? The ethics indicate consent from MNO citizens, and ethical approval from UofT, but there is no indication of MNO involvement in this study, other than mining data from MNO. It appears several authors are affiliated with MNO so likely this study is connected in some way to MNO. It is important to add details to the methods to describe the community level approval for this study, how MNO is directing this research, assuming this study is being done by or at the request of MNO, rather than imposed on MNO by external researchers, and how Indigenous research ethics, such as TCPS 2 Chapter 9, Métis data governance principles, CARE principles, OCAS, and any other relevant policies are being achieved through this study. Is there a data stewardship agreement between MNO and ICES or UofT? | Thank you for this comment so we could include this important context. We have added detail on the involvement of the MNO in the study in the ethics section.  There is a data governance and sharing agreement in place between the MNO and ICES that is described in the manuscript. We have added that the MNO approves all projects under this agreement first. |
| 3. Details of engagement of MNO Elders, executive, government, or other forms of advisors more specifically involved in directing this study should also be provided to highlight the community leadership in this project. | This has been added to our ethics section. |
| 4. It is custom to report numbers only once per manuscript. If rates and variability are reported in a figure, the same numbers should not be reported in the text paragraph referring to the figure. Statements about the trends identified, differences observed, etc. can be made in text, but the specific rates should not be repeated in both locations. | Results have been revised. |
| 5. It might be useful in Figure 1 to map or colour bar the time segments of the lockdown, particularly in reference to the provincial mandates in 2020 and 2021. | Figure 1 has been revised. |
| 6. Discussion: how much greater were the rates of MHA utilization among Ontario youth and general Canadian population? Reporting the specific increases reported in these other studies will aid in understanding the context of increase reported in the present study. | Additional information has been added to the discussion. |
| 1. Introduction line 64: “the Métis” in reference to Métis People collectively would be better termed as “Métis”, similarly to how a phrase would be worded as “Canadians are one of three nations of people in North America” rather than “the Canadians are one of three…” | Thank you for the suggestion. It has been revised accordingly. |
| 2. Introduction line 68-69: the statement is worded as MNO is the only government representing MNO citizens…, which seems overly intuitive/expected. I suspect you are trying to say MNO is the only government representing Métis People in Ontario. Perhaps you are intending to be cognizant that MNO does not represent all Métis in Ontario, in which case this statement might be reworded as MNO is the only government representing Métis People in Ontario, and specifically represents Métis People who have registered as citizens of MNO. | Line 68-69 has been revised accordingly. |
| 3. Anticipating this may change in post-production, figures 2 and 4 are blurry in the review version. | Thank you for the suggestion. Figure 2 and 4 have been updated. |

| Reviewer #2’s comments | Responses |
| --- | --- |
| **Title and Abstract**   - Please describe which variables were statistically adjusted for | Our findings were adjusted for age and sex, and this has now been stated in the abstract. |
| - Consider describing the disaggregated monthly visit rates for mental health conditions (e.g., mood disorders, schizophrenia, substance use disorder etc) even if they did not reach statistical significance. Otherwise, at least mention in the abstract that you did in fact look at the disaggregated mental disorders | Disaggregated monthly visit rates for mental health conditions have been added to the abstract. |
| - The term ‘mental wellness’ which is in the title is used sparingly throughout the manuscript. I recommend describing what ‘mental wellness’ means in Métis cultures and why this terms was used as opposed to other terms (e.g., mental health, mental well-being, social and emotional well-being etc.) | Thank you for this comment. Mental wellness is captured as part of this overall study which used mixed methods, including speaking with MNO citizens and their broader and wholistic understandings wellness which we had inadvertently carried into this paper which is limited to mental health and addiction-related outpatient visits. We have removed mental wellness from this manuscript. |
| - Please ensure keywords are MeSH terms | The keywords have been revised to align with MeSH terminology. The revised keywords are: Indigenous Peoples; COVID-19; Mental Health; Pandemics; Outpatients; Health Care Utilization. |
| **Introduction**   - In the first sentence, I would clarify whether the increased psychological distress sustained since the pandemic, or whether these mental health challenges were only temporary and have since returned to pre-pandemic levels. Indeed, there is research to suggest that mental health in the general public worse in the early course of the pandemic as opposed to the later course (perhaps due to increased awareness, increased vaccination rates, loosening of social restrictions). | The first paragraph of introduction has been revised accordingly. |
| - Line 51. I think it is a large jump to state that Indigenous people’s mental health worsened during the pandemic due to the enduring effects of colonialism. Indigenous peoples are more likely to live in rural and remote locations where they had limited access to delivery services (key during the pandemic), limited access to credit/debit cards (where some stores were refusing to accept cash), and limitations on culturally important social gatherings. While it is important to contextualize the impacts of colonialism, the authors must appropriately attribute what worsened Indigenous people’s mental health during the pandemic | Thank you for the comment. We have revised the text to more clearly distinguish between pandemic-specific factors and the broader historical and structural context. |
| - Line 85. The authors need to strengthen their justification for why Métis-specific data are required | We have added in citations for the need for distinctions-based reporting and linked it to what can happen when Pan-Indigenous approaches are used. |
| **Methods**   - Please justify why 2017-2022 was chosen as the year range | A rationale for this year range was included in study design. |
| **Results**   - The ‘missing’ category in your table 1 should be in a consistent row (either the first or last row of each category) | The missing category in table 1 have been moved to the last row of each category. |
| - Remove the extra em dash in ‘1-Lowest’ | The dash has been removed in table 1. |
| - In the caption, I would remind the readers what ‘Other’ diagnosis entails since it is the second highest category | Thank you for the comment. We have clarified the definition of ‘Other’ diagnosis in the figure 4 caption by explicitly listing the included conditions. |
| - If possible, I would recommend that the researchers further disaggregate the ‘Other diagnosis’ category. It was the second most common category following mood disorders, and it actually had the highest monthly visit rate. The mental disorders in the ‘Other’ category are so different that it’s hard to draw any logical conclusions about what the increased rates mean. Even if all the disorders can’t be disaggregated due to low cell counts, consider reporting the ones that can at least (e.g., trauma-related disorders) | The other diagnosis category has been disaggregated in table 1. |
| **Discussion**   - The discussion requires more work to bring it to a publishable condition. For example, one of the authors’ main findings was that older adult females generally had the highest monthly visit use. However, this finding is scarcely described and justified in the introduction. Why were females more affected and why specifically older adult females? Do older adult females play any important roles in the Métis cultures that can make them more vulnerable to the social and emotional consequences of COVID-19? The discussion needs strengthening overall. While some relevant studies are cited, there is limited critical analysis and comparison of the authors’ findings compared to the extant literature. The discussion requires significantly more enriching discussion of theory, justification, and hypotheses as to why the authors found what they found. Please see my additional comments below | Additional interpretation for the higher rates observed among female and older MNO citizens have been added in the discussion section. |
| - In all of your findings, older adult females had the highest monthly visit use in all categories, except in the ‘Other’ diagnosis where older adult males had the largest increase. Why do you think this is? Also, this finding is another reason why I strongly recommend the authors to further disaggregate the ‘Other’ category and look at each disorder separately (e.g., trauma related disorders vs. OCD vs. personality disorders vs. intentional self-injury) | We have disaggregated the other diagnosis in table 1 and have additional information in the discussion to explain why older adults have higher increase in visit rates in this category. |
| - Line 250 requires a reference | In-text citation has been added to that line. |
| - Line 253. Consider replacing developed countries with high-income countries | Thank you for the suggestion. It has been revised. |
| - I would be careful in using the word ‘Canadians’ when you’re trying to refer to non-Indigenous peoples. While there are some Indigenous peoples that do not refer to themselves as Canadians, this is not the case for all. In consultation with the MNO, I would recommend using the term ‘non-Indigenous peoples’ when trying to compare your findings with the general public. | The reviewer’s caution is noted. We carefully examined where we used “Canadians” and in these contexts, it was not in comparison to Indigenous Peoples or referencing specifically non-Indigenous Peoples but full population-based surveys which would have included both Indigenous and non-Indigenous Peoples and not reported on any comparisons. In this case our study team believes we are appropriately using this term. |
| - Line 275, please explain why you think there were lower than expected visit rates for MNO citizens aged 29 and younger for substance use and higher than expected for older adult females | We have added in some possible explanations for these findings. |
| - Line 287, in this paragraph you must talk about why you think rates were higher in older adult males (and not females like the rest of the findings) | Additional explanation has been added to clarify why visit rates for schizophrenia and other psychotic disorders are higher among males than females. |
| - Line 312 and 314. The in-text citations are a different font/size than the rest of the manuscript. Please correct. | The font and font size of these in-text citations have been updated. |
| - Line 319. In your methods please state what percent of the Métis population is registered with the MNO. | This is not possible to report. The only possible denominator available would be self-reported Métis individuals (e.g. on Canadian Census); however, not all of these individuals would meet the national definition of Métis that the MNO has adopted. The only way to do this would be to get the number of citizens registered to other Métis governments living in Ontario which is not widely reported. |
| - More discussion is required about what policymakers should do with these findings. | We added some additional discussion for this in our concluding paragraph. |
| - The references are different font and size than the rest of the manuscript | The reference list has been changed to the same font and size of the rest of the manuscript. |
