# Supplementary material for: Impact of the COVID-19 pandemic on mental health and addictions-related visits among citizens of the Métis Nation of Ontario: A repeated cross-sectional study
Source: PLOS Ment Health. 2026 Jul 14;3(7):e0000650. doi: 10.1371/journal.pmen.0000650 (PMC13367706; doi:10.1371/journal.pmen.0000650)
Supplement: Peer review history

# Peer review: Decision Letter 0

**DOI:** 10.1371/journal.pmen.0000650.r001

**Authors:** Karli Montague-Cardoso

19 Mar 2026

PMEN-D-25-00534

Impact of the COVID-19 pandemic on mental wellness among citizens of the Métis Nation of Ontario: a repeated cross-sectional study

PLOS Mental Health

Dear Dr. Tsui,

Thank you for submitting your manuscript to PLOS Mental Health and I am sorry for the severe delay in reaching a decision. This was due to difficulties securing reviewers. After careful consideration of the reviewer reports, which we have now received, we feel that your paper has merit but does not fully meet PLOS Mental Health’s publication criteria as it currently stands. Therefore, we invite you to submit a revised version of the manuscript that addresses the points raised during the review process.

Please address all of the points raised, which you can find at the end of this email and in the attached document.

Please submit your revised manuscript by May 03 2026 11:59PM. If you will need more time than this to complete your revisions, please reply to this message or contact the journal office at mentalhealth@plos.org. When you're ready to submit your revision, log on to https://www.editorialmanager.com/pmen/ and select the 'Submissions Needing Revision' folder to locate your manuscript file.

Please include the following items when submitting your revised manuscript:

- A letter that responds to each point raised by the editor and reviewer(s). You should upload this letter as a separate file labeled 'Response to Reviewers'.
- A marked-up copy of your manuscript that highlights changes made to the original version. You should upload this as a separate file labeled 'Revised Manuscript with Track Changes'.
- An unmarked version of your revised paper without tracked changes. You should upload this as a separate file labeled 'Manuscript'.

Guidelines for resubmitting your figure files are available below the reviewer comments at the end of this letter.

We look forward to receiving your revised manuscript.

Kind regards,

Karli Montague-Cardoso

Staff Editor

PLOS Mental Health

**Journal Requirements:**

1. We noticed you have some minor occurrence of overlapping text with the following previous publication(s), which needs to be addressed:

- DOI 10.3389/978-2-8325-3976-7

- doi:10.1001/jamapediatrics.2021.6298

In your revision ensure you cite all your sources (including your own works), and quote or rephrase any duplicated text outside the methods section. Further consideration is dependent on these concerns being addressed.

2. Please amend your detailed Financial Disclosure statement. This is published with the article. It must therefore be completed in full sentences and contain the exact wording you wish to be published.

i. State the initials, alongside each funding source, of each author to receive each grant. For example: "This work was supported by the National Institutes of Health (####### to AM; ###### to CJ) and the National Science Foundation (###### to AM)."

ii. State what role the funders took in the study. If the funders had no role in your study, please state: “The funders had no role in study design, data collection and analysis, decision to publish, or preparation of the manuscript.”

3. Please ensure that your Ethics Statement is available in its entirety at the beginning of your Methods section, under a subheading 'Ethics Statement'.

4. Please provide separate figure files in .tif or .eps format.

For more information about figure files please see our guidelines:

https://journals.plos.org/mentalhealth/s/figures

https://journals.plos.org/mentalhealth/s/figures#loc-file-requirements

5. We have noticed that you have uploaded Supporting Information files, but you have not included a list of legends. Please add a full list of legends for your Supporting Information files after the references list.

6. For studies involving third-party data, we encourage authors to share any data specific to their analyses that they can legally distribute. PLOS recognizes, however, that authors may be using third-party data they do not have the rights to share. When third-party data cannot be publicly shared, authors must provide all information necessary for interested researchers to apply to gain access to the data. (https://journals.plos.org/plosone/s/data-availability#loc-acceptable-data-access-restrictions)

For any third-party data that the authors cannot legally distribute, they should include the following information in their Data Availability Statement upon submission:

1) A description of the data set and the third-party source

2) If applicable, verification of permission to use the data set

3) Confirmation of whether the authors received any special privileges in accessing the data that other researchers would not have

4) All necessary contact information others would need to apply to gain access to the data

If the reviewer comments include a recommendation to cite specific previously published works, please review and evaluate these publications to determine whether they are relevant and should be cited. There is no requirement to cite these works unless the editor has indicated otherwise.

**Additional Editor Comments (if provided):**

[Note: HTML markup is below. Please do not edit.]

Reviewers' comments:

Reviewer's Responses to Questions

**Comments to the Author**

1. Does this manuscript meet PLOS Mental Health’s publication criteria? Is the manuscript technically sound, and do the data support the conclusions? The manuscript must describe methodologically and ethically rigorous research with conclusions that are appropriately drawn based on the data presented.

Reviewer #1: Partly

Reviewer #2: Yes

**********

2. Has the statistical analysis been performed appropriately and rigorously?

Reviewer #1: Yes

Reviewer #2: I don't know

**********

3. Have the authors made all data underlying the findings in their manuscript fully available (please refer to the Data Availability Statement at the start of the manuscript PDF file)?

The PLOS Data policy requires authors to make all data underlying the findings described in their manuscript fully available without restriction, with rare exception. The data should be provided as part of the manuscript or its supporting information, or deposited to a public repository. For example, in addition to summary statistics, the data points behind means, medians and variance measures should be available. If there are restrictions on publicly sharing data—e.g. participant privacy or use of data from a third party—those must be specified.

Reviewer #1: Yes

Reviewer #2: Yes

**********

4. Is the manuscript presented in an intelligible fashion and written in standard English?

PLOS Mental Health does not copyedit accepted manuscripts, so the language in submitted articles must be clear, correct, and unambiguous. Any typographical or grammatical errors should be corrected at revision, so please note any specific errors here.

Reviewer #1: Yes

Reviewer #2: Yes

**********

5. Review Comments to the Author

Please use the space provided to explain your answers to the questions above. You may also include additional comments for the author, including concerns about dual publication, research ethics, or publication ethics. (Please upload your review as an attachment if it exceeds 20,000 characters)

**Reviewer #1:** This manuscript includes an important study of mental health services visits prior to and during the COVID-19 pandemic among Métis people in Ontario, including strong statistical analysis with sex and age specific comparisons.

Below are a few comments for consideration in strengthening this manuscript:

1. Abstract: lines 33-35 and 35-36 repeat the same findings of sex and age disparities in MHA visits.

2. How is this study being conducted in relation to MNO? The ethics indicate consent from MNO citizens, and ethical approval from UofT, but there is no indication of MNO involvement in this study, other than mining data from MNO. It appears several authors are affiliated with MNO so likely this study is connected in some way to MNO. It is important to add details to the methods to describe the community level approval for this study, how MNO is directing this research, assuming this study is being done by or at the request of MNO, rather than imposed on MNO by external researchers, and how Indigenous research ethics, such as TCPS 2 Chapter 9, Métis data governance principles, CARE principles, OCAS, and any other relevant policies are being achieved through this study. Is there a data stewardship agreement between MNO and ICES or UofT?

3. Details of engagement of MNO Elders, executive, government, or other forms of advisors more specifically involved in directing this study should also be provided to highlight the community leadership in this project.

4. It is custom to report numbers only once per manuscript. If rates and variability are reported in a figure, the same numbers should not be reported in the text paragraph referring to the figure. Statements about the trends identified, differences observed, etc. can be made in text, but the specific rates should not be repeated in both locations.

5. It might be useful in Figure 1 to map or colour bar the time segments of the lockdown, particularly in reference to the provincial mandates in 2020 and 2021.

6. Discussion: how much greater were the rates of MHA utilization among Ontario youth and general Canadian population? Reporting the specific increases reported in these other studies will aid in understanding the context of increase reported in the present study.

Minor changes

1. Introduction line 64: “the Métis” in reference to Métis People collectively would be better termed as “Métis”, similarly to how a phrase would be worded as “Canadians are one of three nations of people in North America” rather than “the Canadians are one of three…”

2. Introduction line 68-69: the statement is worded as MNO is the only government representing MNO citizens…, which seems overly intuitive/expected. I suspect you are trying to say MNO is the only government representing Métis People in Ontario. Perhaps you are intending to be cognizant that MNO does not represent all Métis in Ontario, in which case this statement might be reworded as MNO is the only government representing Métis People in Ontario, and specifically represents Métis People who have registered as citizens of MNO.

3. Anticipating this may change in post-production, figures 2 and 4 are blurry in the review version.

**Reviewer #2:** attached. accept with major revisions. attached. accept with major revisions. attached. accept with major revisions. attached. accept with major revisions. attached. accept with major revisions. attached. accept with major revisions.

**********

6. PLOS authors have the option to publish the peer review history of their article (what does this mean?). If published, this will include your full peer review and any attached files.

**Do you want your identity to be public for this peer review?**  If you choose “no”, your identity will remain anonymous but your review may still be made public.

For information about this choice, including consent withdrawal, please see our Privacy Policy.

Reviewer #1: No

Reviewer #2: No

**********

[NOTE: If reviewer comments were submitted as an attachment file, they will be attached to this email and accessible via the submission site. Please log into your account, locate the manuscript record, and check for the action link "View Attachments". If this link does not appear, there are no attachment files.]

-->Figure Resubmissions:

While revising your submission, we strongly recommend that you use PLOS’s NAAS tool (https://ngplosjournals.pagemajik.ai/artanalysis) to test your figure files. NAAS can convert your figure files to the TIFF file type and meet basic requirements (such as print size, resolution), or provide you with a report on issues that do not meet our requirements and that NAAS cannot fix.

After uploading your figures to PLOS’s NAAS tool - https://ngplosjournals.pagemajik.ai/artanalysis, NAAS will process the files provided and display the results in the "Uploaded Files" section of the page as the processing is complete. If the uploaded figures meet our requirements (or NAAS is able to fix the files to meet our requirements), the figure will be marked as "fixed" above. If NAAS is unable to fix the files, a red "failed" label will appear above. When NAAS has confirmed that the figure files meet our requirements, please download the file via the download option, and include these NAAS processed figure files when submitting your revised manuscript.

# Author comment: Author response to Decision Letter 1

**DOI:** 10.1371/journal.pmen.0000650.r002

31 May 2026

Attachment Submitted filename: Response table 20260526.docx

# Editor report: Decision Letter 1

**DOI:** 10.1371/journal.pmen.0000650.r003

**Authors:** Karli Montague-Cardoso, Karli Montague-Cardoso

16 Jun 2026

Impact of the COVID-19 pandemic on mental health and addictions-related visits among citizens of the Métis Nation of Ontario: a repeated cross-sectional study

PMEN-D-25-00534R1

Dear Ms. Tsui,

We are pleased to inform you that your manuscript 'Impact of the COVID-19 pandemic on mental health and addictions-related visits among citizens of the Métis Nation of Ontario: a repeated cross-sectional study' has been provisionally accepted for publication in PLOS Mental Health.

Before your manuscript can be formally accepted you will need to complete some formatting changes, which you will receive in a follow up email. A member of our team will be in touch with a set of requests.

Please note that your manuscript will not be scheduled for publication until you have made the required changes, so a swift response is appreciated.

IMPORTANT: The editorial review process is now complete. PLOS will only permit corrections to spelling, formatting or significant scientific errors from this point onwards. Requests for major changes, or any which affect the scientific understanding of your work, will cause delays to the publication date of your manuscript.

If your institution or institutions have a press office, please notify them about your upcoming paper to help maximize its impact. If they'll be preparing press materials, please inform our press team as soon as possible -- no later than 48 hours after receiving the formal acceptance. Your manuscript will remain under strict press embargo until 2 pm Eastern Time on the date of publication. For more information, please contact mentalhealth@plos.org.

Thank you again for supporting Open Access publishing; we are looking forward to publishing your work in PLOS Mental Health.

Best regards,

Karli Montague-Cardoso

Staff Editor

PLOS Mental Health

***********************************************************

Reviewer Comments (if any, and for reference):
